# Morphological, Structural, Thermal, Pasting, and Digestive Properties of Starches Isolated from Different Varieties of Rice: A Systematic Comparative Study

**DOI:** 10.3390/foods12244492

**Published:** 2023-12-15

**Authors:** Xiaojun Lin, Xuanyi Zhang, Bin Du, Baojun Xu

**Affiliations:** 1 Food Science and Technology Program, Department of Life Sciences, BNU-HKBU United International College, Zhuhai 519087, China; e1124349@u.nus.edu (X.L.); q030013039@mail.uic.edu.cn (X.Z.); 2 Hebei Key Laboratory of Natural Products Activity Components and Function, Hebei Normal University of Science and Technology, Qinhuangdao 066004, China; bindufood@aliyun.com

**Keywords:** rice starch, gelatinization, retrogradation, starch digestibility

## Abstract

The aim of this study was to compare the properties of isolated starches from ten commonly consumed rice varieties in China and to investigate their possible association. In addition, principal component analysis (PCA) and correlation analysis were performed to demonstrate the weight or relevance of different properties. The starch granules had an irregular polyhedral structure. The crystalline structure had an orthogonal arrangement, which is characteristic of A-type starch with nanocrystals with an orthorhombic crystal structure. In addition, higher levels of rapidly digestible starch (72.43 to 74.32%) and resistant starch (2.27 to 2.3%) were found in glutinous rice starch. The highest content of slowly digestible starch (59.48%) was found in starch isolated from black rice, which may be an ideal rice variety for controlling blood glucose and weight. Starch isolated from red Hani terrace rice showed the highest thermal stability during cooking and the highest resistance to a high shear force treatment. In addition, the PCA suggests that the amylose content of starch largely determines the functional properties of starch and positively correlates with the peak viscosity and setback viscosity of the starch pasting. The results of this study will enrich the scientific knowledge of various rice starches and promote their application in the food industry and other industries.

## 1. Introduction

Starch is a micro- or submicron-sized particle that has amylose and amylopectin as its main constituents [1]. Amylose is a linear polymer made predominantly of D-glucose units linked by α-D-(1-4) glycosidic bonds [1]. In contrast, amylopectin is a highly branched molecule, where its main chain and branches are connected through α-D-(1-4) linkages, with branching occurring at α-D-(1-6) linkage points [1]. Beyond its nutritional value, starch finds diverse applications in the food industry as a stabilizing, gelling, emulsifying, and thickening agent. It also serves as a substrate in the production of sweeteners and ethanol. Furthermore, starch extends its utility to non-food sectors, functioning as a dusting powder in cosmetics, an aid in papermaking, and as an excipient in the formulation of pharmaceutical tablets [2].

The majority of commercial starches are separated from dietary staples including corn, rice, wheat, and potatoes. Rice (*Oryza sativa* L.) is a cereal widely consumed worldwide, serving as the main source of human food. As the largest consumer and producer of rice in the world, China produced 154.9 million metric tons in 2021 [3]. Rice starch, when contrasted with starches derived from other cereals such as corn and wheat, demonstrates a broader spectrum of physicochemical properties and functionalities. It is characterized by its mild and creamy taste, hypoallergenic nature, and digestibility [4]. Additionally, rice starch exhibits notable freeze–thaw stability in its paste form and possesses robust resistance to acidic conditions [4]. These unique properties increase consumer acceptance and demand for rice starch in the food industry.

The starches isolated from various rice cultivars have a great diversity in their granular size and shape, as they have various genetic backgrounds, as well as climatic and soil conditions. The physicochemical characteristics of starch are largely associated with the texture and nutritional attributes of rice starch products [5].

Rice, a staple food globally, is commonly prepared by cooking its grains or flour derivatives in water. This process involves the irreversible swelling of starch granules as they are heated in an aqueous environment [1]. Notably, when starch is heated within the temperature range of 60 °C to 70 °C, its insoluble granules undergo disintegration due to the applied thermal energy. This disintegration leads to molecular disarray and the subsequent loss of the crystalline structure, a phenomenon referred to as gelatinization [1]. Gelatinization transforms starch into the physical form required by many food systems; however, starch is thermodynamically unstable and undergoes changes that affect its technical suitability [6]. Subsequent to the gelatinization phase, a cooling process initiates the reorganization of starch chains into a more ordered structure [7]. This phenomenon, termed retrogradation, is characterized by the reassociation of starch molecules [7]. The distinct behaviors of starch during gelatinization and retrogradation serve as reliable indicators in determining its suitability as an ingredient or additive in various food products and industrial applications [8].

Understanding the pasting properties and digestive characteristics of starch is essential to study the characteristics of starch and to propose new applications of starch in industries. Different rice varieties tend to exhibit different pasting properties; consequently, it is necessary to consider the type of starch used, with a view to determining the properties of end products. So far, there have been few systematic comparative studies on the characteristics of starch from different rice sources. Hence, the aim of this study is to determine the physiochemical properties of starch isolated from different varieties of rice consumed in China and to investigate its potential applications. 

## 2. Materials and Methods

### 2.1. Rice Samples

**Figure 1:**
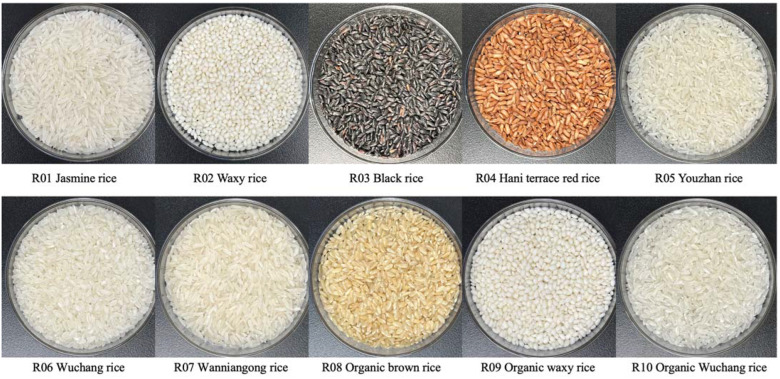
Morphology of different varieties of rice.

Rice samples of 10 varieties were obtained from different regions of China, purchased from agricultural companies specializing in crop production. Table 1 illustrates the attributes of rice samples, and the morphology of rice grains is displayed in Figure 1.

### 2.2. Starch Isolation from Rice Samples

Starch was extracted by following the procedures in a previous study [9]. The rice was immersed in distilled water for 12 h and then ground into a slurry. The slurry was mixed with 0.2% NaOH in a volume ratio of 1:5 for 48 h. The starch precipitate was sieved by a 100-mesh sieve. After sieving, the starch was settled to remove supernatants. Subsequently, the starch precipitated 3 times after the addition of water. The starch samples were freeze-dried at −80 °C for 24 h, and dry powder was obtained.

### 2.3. Moisture Content Determination

A moisture analyzer (HF37, Mettler Toledo, OH, USA) was applied to determine moisture contents of starch. Starch was laid flat on a tray, and the analyzer heated the starch to calculate the lost moisture. Moisture content was determined by measuring the weight loss of a sample when heated, assuming the loss is due to moisture evaporation.

### 2.4. Color Attributes Analysis

Color attributes of L*, a*, and b* of isolated starches were measured by a colorimeter (CR-400, Konica Minolta, Tokyo, Japan). Color values of L*, a*, and b* of native and pregelatinized starches were measured by a colorimeter. L* is a measure of lightness on a scale from 0 (black) to 100 (white). The a* value indicates the position between red (positive values) and green (negative values) in the color spectrum, while b* measures the balance between yellow (positive values) and blue (negative values).

### 2.5. Morphological Analysis on the Microstructure of Starch

The morphological attribute of starch was analyzed using a scanning electron microscope (SEM, MIRA3, TESCAN, Sydney, Australia) and operated under a 5 kV accelerating voltage. The magnification was 5000×, and the unit length of the image display was 1 μm. A small quantity of each freeze-dried starch sample was carefully attached to an aluminum specimen holder using double-sided adhesive tape and then meticulously sputter-coated with gold in a vacuum environment. A representative number of starch granules were selected randomly from different areas within each image. Using image analysis software (ImageJ v 1.48), the dimensions of each selected granule were measured against the provided scale bar. The same dimension (the longest axis) was measured for each granule. The average size of the granules was then calculated by taking the mean of these measurements.

### 2.6. X-ray Diffraction Analysis

The X-ray diffraction (XRD) pattern of the isolated starches’ freeze-dried powder was determined by an X-ray diffractometer (Ultima IV, Rigaku, Tokyo, Japan) at a target of 40 kV voltage and 40 mA current with irradiation from Cu, Ka, and a wavelength of 0.1542 nm. The diffraction angle (2*θ*) was scanned from 3° to 40°. The crystallinity degree (CD) was calculated according to Nara and Komiya [10] by employing ORIGIN 2021 (Microbial, Northampton, MA, USA):

$$CD\;\left(\%\right)=\frac{Ac}{Ac+Aa}\times 100\%$$

where *Ac* and *Aa* correspondingly represent the crystalline and amorphous areas.

### 2.7. Determination of Apparent Amylose Content

The amylose content was analyzed by an amylose content analyzer (DPCZ-II, Daji Electric Instrument Co., Ltd., Hangzhou, China). Before analysis, 0.1 g of starch was boiled for 10 min with the addition of 1 mL of anhydrous ethanol and 9 mL of 1 M sodium hydroxide. The cooled sample was diluted into 100 mL. After degreasing with petroleum ether, the sample was reacted with an iodine reagent. After the sample reacted with the reagent to develop color for 10 min, it was loaded into the analyzer. The amylose content was determined by configuring amylose standard solution in gradient concentrations and plotting a standard curve of absorbance and concentration.

### 2.8. Determination of Swelling Power and Water Solubility Index

The swelling power (SP) and water solubility index (WSI) were based on procedures outlined by Guo et al. [11]. In a test tube, 2% (*w*/*v*) starch solution in 15 mL was heated at 90 °C for 30 min while being shaken. The cooled starch paste was centrifugated at 3000× *g* for 30 min. The obtained supernatant was evaporated at 105 °C in an air oven until constant weight. The SP and WSI of each sample were determined three times. SP and WSI were calculated using the following formulas:

$$SP\left(g/100g\right)=\frac{Weight\;of\;sediment}{Weight\;of\;dry\;sample\times \left(100-water\;solubility\;index\right)}\times 100$$

$$WSI\left(g/100g\right)=\frac{Weight\;of\;total\;solids\;in\;supernatant}{Weight\;of\;dry\;sample\;solids}\times 100$$

### 2.9. Pasting Properties Analysis

Pasting profiles were determined by a Rapid Visco Analyzer (RVA 4500, Perten, Warriewood, Australia). In the RVA can, 3.5 g of starch and 25 mL of distilled water were combined. Following a 60 s holding period at 50 °C, the temperature rose at a rate of 6 °C per minute. The temperature was maintained at 95 °C for 5 min before falling by 6 °C each minute. During the last 2 min, the temperature was held at 50 °C. The viscosity of the samples was measured at different temperatures. The temperature corresponding to the first significant increase in viscosity refers to the pasting temperature (PT). The highest viscosity reached with heating refers to the peak viscosity (PV) and the minimum viscosity refers to the trough viscosity (TV). The difference between PV and TV is the breakdown viscosity (BD). As the sample cools, the viscosity increases to the final viscosity. The difference between the final viscosity and the minimum viscosity is the setback viscosity (SB).

### 2.10. Thermal Analysis

Gelatinization properties of starch were measured using a Differential Scanning Calorimeter (DSC, Mettler-Toledo Inc., Columbus, OH, USA) based on the method outlined by Guo et al. [11]. A 3 mg dry basis starch and distilled water (7 μL) were mixed in a DSC pan. The measured temperature rose from 20 °C to 120 °C at a rate of 10 °C/min. The gelatinization temperatures of isolated starch, including the onset temperature (T_o_), peak temperature (T_p_), conclusion temperature (T_c_), and enthalpy of gelatinization (ΔH) were determined.

### 2.11. Thermogravimetric Analysis

The thermogravimetric analysis (TGA) was performed with a thermal gravimetric analyzer (STA 409 PC/PG, Netzsch, Weimar, Germany). A small aluminum oxide crucible was used to contain the 2–3 mg sample. The temperature range was from 20 °C to 760 °C, with a heating rate of 10 °C/min and a nitrogen flow rate of 30 mL/min.

### 2.12. In Vitro Digestibility Analysis

The in vitro digestibility of starch was analyzed based on a previous method [12], with modifications. Porcine pancreatin (P7545, Sigma-Aldrich, St. Louis, MI, USA) (0.03 g) was dispersed in 100 mL sodium maleate buffer (0.1 M, pH 6.0) with 10 min stirring. Subsequently, the dispersion went through 10 min centrifugation at 3000× *g* to obtain the supernatant. Amyloglucosidase (A800618, Macklin, Shanghai, China) (1 mL) was diluted using 10 mL sodium maleate buffer. Diluted amyloglucosidase (1 mL) was mixed with the supernatant of pancreatin to obtain the enzyme solution.

Starch (0.1 g, dry basis) was added into 10 mL buffer with homogenization and heated in a boiling water bath for 30 min. After heating, the starch solution (37 °C) was mixed with five glass balls and 4 mL of the fresh enzyme solution. After homogenization, the starch was incubated at 37 °C in a water bath with continuous agitation. The 0.5 mL of hydrolysate was added into 4.5 mL of 80% ethanol after 20 min and 120 min of digestion. The 3,5-dinitrosalicylic acid (DNS) method was used to determine the amount of glucose.

Based on the glucose content after 20 min and 120 min digestion, the proportions of rapidly digestible starch (RDS), slowly digestible starch (SDS), and resistant starch (RS) were calculated based on the following formulas:

$$\mathrm{RDS}\;(\%)=\frac{(G20\text{-}\mathrm{FG})\text{×}0.9}{\mathrm{TS}}\text{×}100$$

$$\mathrm{SDS}\;(\%)=\frac{(G120\text{-}G20)\text{×}0.9}{\mathrm{TS}}\text{×}100$$

$$\mathrm{RS}\;(\%)=\frac{\left(\mathrm{TS}\text{-}\mathrm{RDS}\text{-}\mathrm{SDS}\right)}{\mathrm{TS}}\text{×}100$$

where *TS* denotes the dry basis of starch, *FG* denotes the free glucose concentration in starch without digestion, and *G*20 and *G*120 denote the glucose content in the starch hydrolysate obtained after 20 min and 120 min digestion, respectively.

### 2.13. Statistical Analysis of Experimental Data

All parameters in the present study were determined three times based on three-times sampling. The variance (ANOVA) of the data was analyzed by SPSS 26 (IBM, Chicago, IL, USA) based on Duncan’s test (*p* < 0.05, *n* = 3). The principal component analysis and Pearson’s correlation analysis were performed on the physicochemical properties of the starches.

## 3. Results

### 3.1. Moisture Content and Color Characteristics of Isolated Starch

Table 3 illustrates the moisture content of the starch samples. The moisture content varied from 3.67% to 9.25%. SOR01 presented the highest (9.25%) moisture content, whereas SOR10 had the lowest moisture content (3.67%).

The color parameters of the isolated starches are illustrated in Table 3. SOR03 showed the lowest lightness (L*) value (94.66), while SOR10 had the highest L* value (99.15). SOR03 presented the highest redness (a*) value (0.86), while SOR01 had the lowest a* value (−1.01). The yellowness (b*) value of SOR03 was maximum (6.14), while that of the b* value of SOR02 was minimum (0.81).

### 3.2. Morphology of Isolated Starch

**Figure 2:**
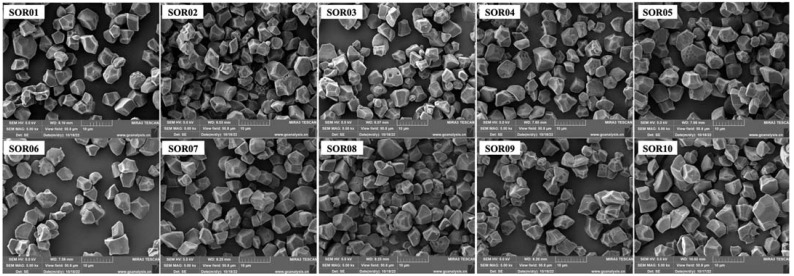
Photographs of starch granules observed by SEM. SOR01, SOR02, SOR03, SOR04, SOR05, SOR06, SOR07, SOR08, SOR09, and SOR10 indicate starch isolated from Jasmine rice, waxy rice, black rice, Hani terrace red rice, Youzhan rice, Wuchang rice, Wanniangong rice, organic brown rice, organic waxy rice, organic Wuchang rice, respectively.

As shown in Figure 2, the microstructural morphology of starch samples was observed by the SEM. The starch granules were irregularly polyhedral in shape with obvious edges. Compared to starch isolated from non-glutinous rice, the surface of starch granules from glutinous rice (SOR2 and SOR9) exhibited more significant depressions and folds. The shapes of the starch particles were similar. The sizes of the starch granules typically ranged between 2.5 and 7.4 μm. However, the starch granules isolated from Wanniangong rice and organic Wuchang rice had an average size of 3.2 to 10.4 μm and 3.5 to 8.4 μm, respectively.

### 3.3. X-ray Diffraction of Isolated Starch

**Figure 3:**
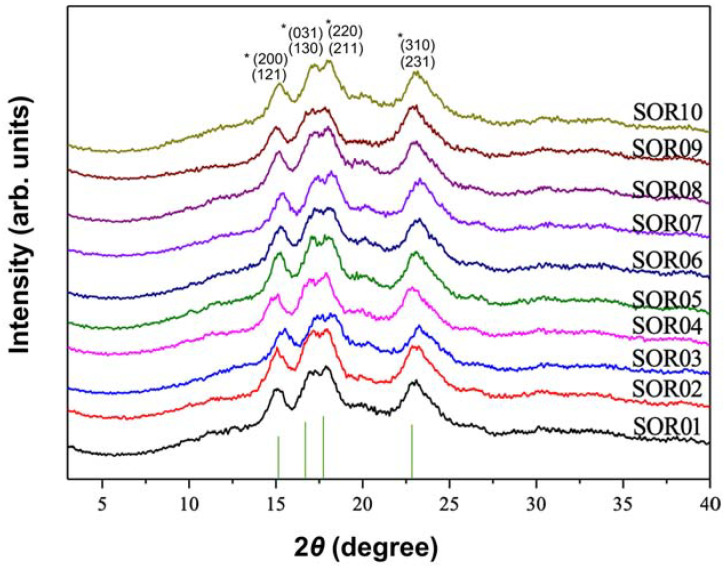
X-ray diffraction patterns of isolated starch. SOR01, SOR02, SOR03, SOR04, SOR05, SOR06, SOR07, SOR08, SOR09, and SOR10 indicate starch isolated from Jasmine rice, waxy rice, black rice, Hani terrace red rice, Youzhan rice, Wuchang rice, Wanniangong rice, organic brown rice, organic waxy rice, and organic Wuchang rice, respectively. * Indicates miller indices (h, k, l).

As Table 2 delineates, the XRD profiles of the rice starch samples (SOR01 through SOR10) exhibited four primary diffraction peaks at specific 2*θ* angles. These peaks, indicative of the crystalline structure, were consistently located around 15.1°, 17.2°, 18.0°, and 23.0°. The corresponding Miller indices, as depicted in Figure 3, elucidate the crystalline orientation of these starches.

For isolated starch, the relative crystallinity varied from 23.82% to 39.65%. The SOR09 had the highest crystallinity, whereas the lowest crystallinity was found in SOR06 (Table 3).

### 3.4. Apparent Amylose Content of Isolated Starch

Table 3 provides a summary of the amylose content of various starches. The amylose contents of starch isolated from waxy rice and organic waxy rice were 1.67% and 2.55%, respectively. For non-glutinous rice, the amylose content of starch from black rice was the highest (26.57%), while that of Hani terrace red rice was the lowest (13.95%).

### 3.5. Swelling Power and Water Solubility Index of Isolated Starch

The SP of isolated starch ranged between 27.69% and 51.32%, the lowest SP for starch from organic brown rice and the highest one for organic waxy rice (Table 3). The starch isolated from waxy rice and organic waxy rice showed no water solubility, whereas organic brown rice showed the highest water solubility index (21.36%).

### 3.6. Pasting Profiles of Isolated Starch

**Figure 4:**
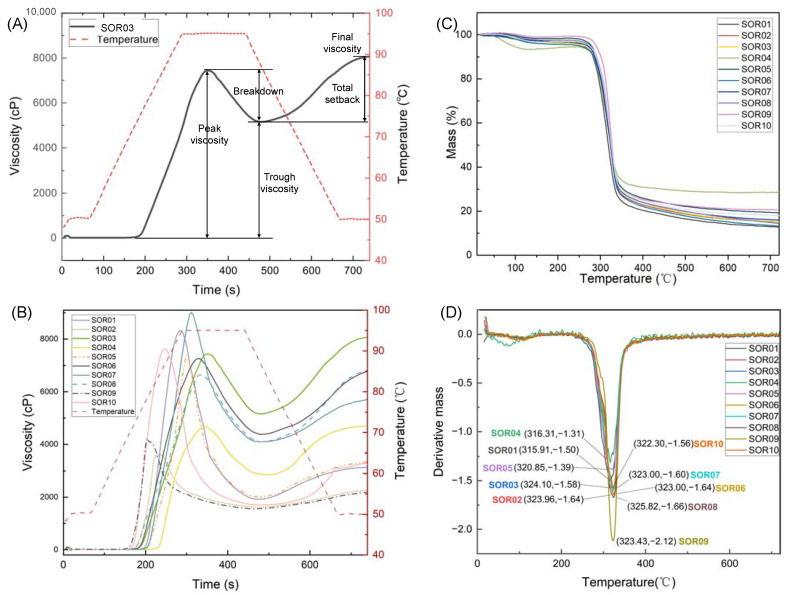
(**A**) A typical RVA pasting profile. SOR03 indicates starch from black rice. (**B**) Pasting profile of different rice starches. (**C**) The thermogravimetric curves of isolated starch. (**D**) The derivative thermogravimetric curve of isolated starch. The lowest value of the derivative mass of each curve and its corresponding temperature (°C) are plotted. Symbols including SOR01, SOR02, SOR03, SOR04, SOR05, SOR06, SOR07, SOR08, SOR09, and SOR10 indicate starch isolated from Jasmine rice, waxy rice, black rice, Hani terrace red rice, Youzhan rice, Wuchang rice, Wanniangong rice, organic brown rice, organic waxy rice, and organic Wuchang rice, respectively.

As displayed in Figure 4B, isolated starches from different varieties of rice exhibit different pasting profiles. The pasting parameters of the isolated starches determined by RVA are interpreted in Figure 4A, and the results are summarized in Table 4. Figure 4B illustrates the trends in viscosity (left vertical axis) and temperature (right vertical axis) over time (horizontal axis). The red dashed line represents the temperature changes throughout the process. The remaining curves depict the viscosity changes of ten different types of rice starch during the process. All starch samples demonstrate a trend where the viscosity initially increases to a peak value, and then it decreases, subsequently rising again. The pasting temperatures (PT) were between 69.43 °C and 83.40 °C, the lowest PT for starch from organic waxy rice (SOR09) and the highest for starch from Hani terrace red rice (SOR04). The starch from waxy rice (SOR02) had the lowest peak viscosity (3603 cP), whereas the starch from Wanniangong rice (SOR07) presented the highest peak viscosity (8998 cP). Trough viscosity (TV) was between 1560 and 5215 cP, the highest for starch from black rice (SOR03) and the lowest for starch from organic waxy rice (SOR09). The breakdown viscosity (BD) varied between 1789 and 6487 cP. The starch from Jasmine rice (SOR01) presented the highest BD, while the starch from Hani terrace red rice (SOR04) displayed the lowest one. The setback viscosity (SB) varied between 625 and 2923 cP, the highest for starch from black rice (SOR04) and the lowest for waxy rice (SOR02). The final viscosity (FV) of starch was between 2204 cP (SOR09) and 8138 cP (SOR04).

### 3.7. Thermogravimetric Properties of Isolated Starch

Figure 4C,D display the thermogravimetric (TG) curves and derivative thermogravimetric (DTG) curves of isolated starch. In Figure 4C, all depicted curves follow a pattern of an initial rapid decline and a subsequent stabilization; this decrease symbolizes the mass loss of starch during heating. This mass loss can be categorized into three distinct phases. In the first phase, spanning from 20 °C to 100 °C, SOR04 exhibits the most significant mass loss at 5.51%, whereas SOR09 demonstrates no loss. Figure 4D presents the changes in DTG (Derivative Thermogravimetry) values across a temperature range of 20 °C to 760 °C, with the minimum value observed at approximately 320 °C. Notably, the lowest DTG value for SOR01 corresponds to a temperature of 315.91 °C, whereas for SOR08, it corresponds to a higher temperature of 325.82 °C. At the conclusion of the heating process, SOR01 shows the highest mass loss at 87.23%, in contrast to SOR04, which records the lowest at 71.38%.

### 3.8. Thermal Properties of Isolated Starch

Table 5 summarizes the onset temperature (T_o_), peak temperature (T_p_), conclusion temperature (T_c_), and enthalpy of gelatinization (ΔH) for starch gelatinization. The lowest onset temperature (55.65 °C), the lowest peak temperature (61.52 °C), and the lowest conclusion temperature (67.89 °C) occurred in starch isolated from organic brown rice (SOR08), while the highest onset temperature (70.61 °C), the highest peak temperature (75.61 °C), and the highest conclusion temperature (81.04 °C) occurred in starch isolated from Hani terrace red rice (SOR04). The enthalpy of gelatinization (ΔH) ranged from 6.74 to 13.91 J/g, the highest value for starch from Hani terrace red rice (SOR04) and the lowest one from organic Wuchang rice (SOR10).

### 3.9. In Vitro Digestibility of Isolated Starch

Under in vitro digestion conditions, the proportion of rapidly digestible starch (RDS), slowly digestible starch (SDS), and resistant starch (SOR) contents are illustrated in Table 5. As shown in Table 5, the SOR of all isolated starches was less than 2.3%, and the SOR of starch isolated from organic and normal waxy rice was higher than the others. The RDS values ranged from 39.80% to 74.32%, the lowest RDS value for the starch from black rice and the highest for starch from waxy rice. The range of SDS values was from 23.41% to 59.48%, the lowest SDS value for the starch from waxy rice and the highest for starch from black rice.

### 3.10. Principal Component and Correlation Analysis of Isolated Starch Properties

**Figure 5:**
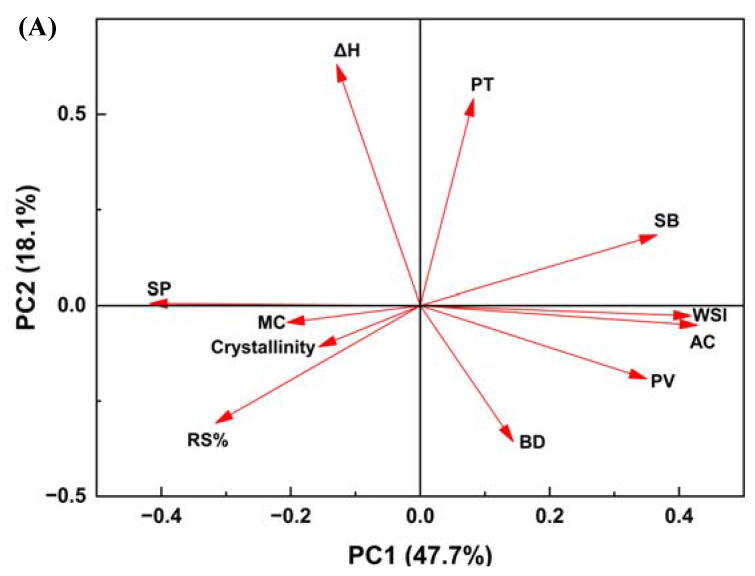

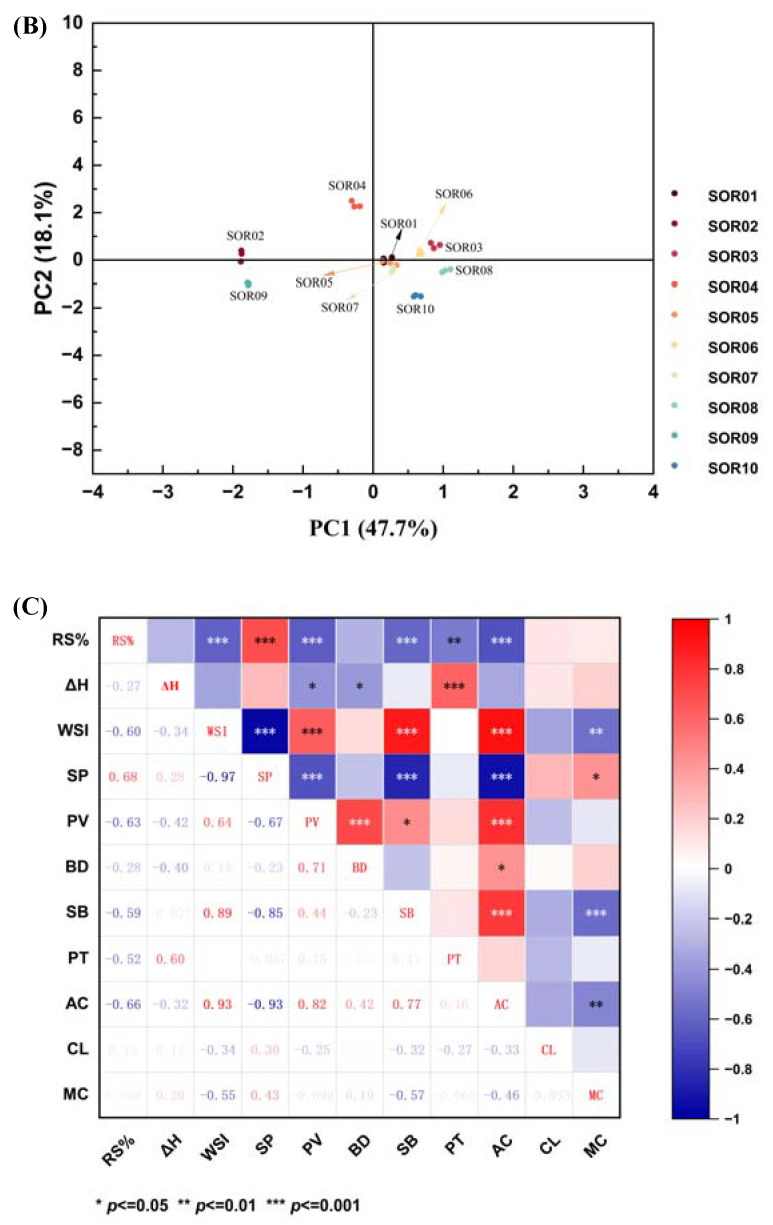
Principal component analysis (**A**), cluster analysis (**B**), and correlation analysis (**C**) based on the pasting, thermal, and digestive properties of different rice starches. Symbols including SOR01, SOR02, SOR03, SOR04, SOR05, SOR06, SOR07, SOR08, SOR09, and SOR10 indicate starch isolated from Jasmine rice, waxy rice, black rice, Hani terrace red rice, Youzhan rice, Wuchang rice, Wanniangong rice, organic brown rice, organic waxy rice, and organic Wuchang rice, respectively. RS%: resistant starch content, ΔH: enthalpy of gelatinization, SP: swelling power, WSI: water solubility index, PV: peak viscosity, BD: breakdown viscosity, SB: setback viscosity, CL: crystallinity.

The results from the principal component analysis (PCA) reveal that the two principal components, PC1 and PC2, effectively encapsulate the primary characteristics of the studied starches, summing up to a notable 65.8% of the total variance (Figure 5A,B). In the loading plot (Figure 5A), the physicochemical properties displayed distinct spatial orientations. Swelling power (SP) exhibited a significant negative alignment with PC1, which accounts for the largest variance among the properties measured. In contrast, water solubility index (WSI) an amylose content (AC) showed a notable positive correlation with the second principal component (PC2). In the scores plot (Figure 5B), there is a noticeable clustering amongst starch from Jasmine, Youzhan, and Wanniangong rice. Conversely, starch from waxy and organic waxy rice exhibit a pronounced separation from the rest.

The correlation heatmap (Figure 5C) delineates the relationships among the physicochemical properties. The amylose content of starch was associated with several parameters, negatively correlated with the swelling power and content of resistant starch (*p* ≤ 0.001). Moreover, the amylose content was positively correlated with the peak viscosity and setback viscosity of starch (*p* ≤ 0.001).

## 4. Discussion

### 4.1. Morphological and Structural Characteristics of Isolated Rice Starches

In X-ray diffraction processes that utilize a singular wavelength of 0.1542 nm, similar to those in powder X-ray diffraction methods, the curve produced from such an analysis is termed an X-ray pattern [13]. The consistency of these peaks across the samples suggests a commonality in the crystalline structure of the starches, with minor variations in peak positions indicating subtle differences in the lattice parameters or crystallinity among the varieties. These variances could be attributed to the intrinsic properties of the starch source [14]. Through a meticulous XRD peak analysis and alignment with referenced Miller indices for orthorhombic crystal structures, the rice starch samples’ diffraction patterns corresponded distinctly to the orthorhombic phase characteristics defined in the amaranth starch standards [15]. This concordance substantiates the presence of an orthorhombic crystalline framework within the rice starch granules, affirming their structural taxonomy and furthering the understanding of starch crystallinity in cereal science. Since starch was isolated from the same botanical source of rice, starch granules exhibited similar granule shapes (irregular polyhedral) but various sizes. Starch isolated from Wanniangong rice and organic Wuchang rice exhibited larger granule sizes than those of other varieties of rice. The size and shape vary from cultivar to cultivar, which are influenced mainly by the germplasm, climatic conditions, and agronomic practices [14]. Moreover, the size of starch granules affects their composition, pasting behaviors, enzyme resistance, and crystallinity [16].

Starches exhibit two distinct crystal structures as revealed by their X-ray diffraction (XRD) patterns. Those with an orthorhombic crystal structure are categorized as A-type starches, while those exhibiting a hexagonal crystal structure are classified as B-type [17]. Additionally, starches that present a combination of both orthorhombic and hexagonal crystal structures, often in the form of nanocrystals, are identified as C-type [17]. In this study, the rice starches that exhibited distinct diffraction peaks are indicative of an orthorhombic crystal structure, which is a characteristic feature of A-type starch commonly found in cereals [15]. A dense accumulation of crystal structures of A-type starch allows for a higher resistance to chemical reactions, such as acid hydrolysis [18].

The relative crystallinity of isolated starches was between 23.82% and 39.65%. The organic waxy rice exhibited the highest crystallinity (39.65%), whereas the normal waxy rice exhibited the lowest crystallinity (23.82%). The crystallinity in starch primarily arises from nanocrystals with orthorhombic and/or hexagonal crystal structures, as noted by Gong et al. [19]. Meanwhile, the lamellar structure represents a partially crystalline formation within starch granules, comprising alternating crystalline and amorphous regions [19]. The amorphous regions mainly contain the less regularly structured branches of amylose [19]. Since amylose contents are negatively associated with the relative crystallinity (RC), the RC of glutinous rice starches was higher than non-glutinous starches [6]. However, the amylose contents of organic and normal waxy rice starch were similar, being 1.67% and 2.55%, respectively, both significantly lower than those of other non-glutinous rice. The differences in crystallinity of starch granules can be caused by several factors, including amylopectin chain length, degree of amylopectin ordering, amylose-lipid complexes, and starch granule size [20,21]. The internal structure of amylopectin clusters can be further studied by enzymatic hydrolysis and size exclusion chromatography [22]. 

### 4.2. Physio-Chemical Properties of Isolated Rice Starches

The color attributes of starch derived from organic Wuchang rice (RS10) suggest its suitability as a kind of food additive in food or other products, attributed to its high lightness and comparatively low levels of redness and yellowness. Additionally, parameters such as swelling power (SP) and water solubility index (WSI) were employed to evaluate the interaction between water molecules and amylopectin chains [23]. The swelling and rise in solubility result from the disruption of hydrogen bonds by heating, allowing for the attachment of water molecules to the revealed hydroxyl groups of starches chains through hydrogen bonds [21]. The starch isolated from the organic waxy rice (SOR09) had the highest SP, followed by the starch isolated from the waxy rice (SOR02) because of their low amylose contents. Previous studies have shown that the swelling is mainly caused by the crystalline structure of amylopectin, mainly a short-chain amylopectin with a degree of polymerization from 6 to 9 [15]. Generally, water solubility indicates the quantity of solubilized starch under a specific temperature [11]. As displayed in Table 3, no significant solubility of SOR09 and SOR02 was observed because of their low amylose content [24]. SOR02 and SOR09 exhibit high swelling characteristics with swelling power values exceeding 50 g/100 g, combined with negligible water solubility indices, indicating a pronounced ability to absorb water and swell without disintegrating, suitable for applications requiring a high viscosity and gel strength [25]. On the other hand, samples SOR01, SOR04, SOR05, and SOR07 display medium swelling properties, with swelling power in the range of approximately 36 to 41 g/100 g. These samples offer a balance between viscosity and stability, making them versatile for a broader range of food products [26]. Sample SOR08 presents a restricted swelling ability, as evidenced by the lowest swelling power value of 27.69 g/100 g and the highest solubility index of 21.36 g/100 g, suggesting that such a starch may be more appropriate for products where a low viscosity is desired and where starch breakdown could contribute to sweetness or texture [27]. Furthermore, starch isolated from organic brown rice (SOR08) demonstrated the highest level of water solubility among the varieties tested. This variation in water solubility is primarily attributed to the distinct distribution patterns of amylopectin within the starch granules. During the heating process, a portion of amylose may escape from these granules, thereby contributing to an increase in the overall solubility of the starch [8]. Finally, samples SOR03, SOR06, and SOR10, while having a lower swelling power, show a higher solubility, indicating a potential for a restricted swelling capacity. These might be suitable for products that require a balance between thickening and water holding without significant gel formation [28].

### 4.3. Thermal Attributes of Isolated Starch

When heated in water, the double helix structure of amylose is disrupted over time, leading to the release of both amylopectin and amylose [8]. The process for these changes is gelatinization, with changes in the viscosity, which occur over a range of temperatures, depending on the different plant sources of the starch [29]. DSC was applied to measure the gelatinization temperatures. T_p_ is a critical parameter representing the temperature at which maximum water absorption and swelling occur, leading to the melting of crystalline regions of starch granules. Various intrinsic and extrinsic factors are known to influence T_p_, among which the amylose–amylopectin ratio, starch granule size, crystallinity, and the presence of lipids and other components are the most significant [30]. 

As shown in Table 5, the highest onset temperature (T_o_) of 70.61 °C was observed for starch isolated from Hani terrace red rice (SOR04), indicating superior thermal stability. This enhanced stability can be attributed to several factors. Firstly, the integrity of the starch crystallites plays a crucial role; fewer defects in the crystalline structure contribute to greater thermal stability [31]. “Crystallite integrity” in starch refers to how free the microscopic crystalline regions, mainly composed of densely packed amylopectin molecules, are from defects. Rice starch crystallites, which have an orthorhombic crystal structure, are particularly notable in this regard. This structure means the starch molecules are arranged in a repeating rectangular-prism pattern, contributing to the starch’s unique properties. A high integrity in these orthorhombic crystallites equates to fewer structural defects, enhancing the starch’s resistance to thermal breakdown. Thus, rice starch with a high crystallite integrity remains stable for a longer duration under heat, delaying the onset of gelatinization or melting. In contrast, starch with more crystalline defects exhibits lower thermal stability, breaking down at lower temperatures [31,32]. Secondly, the unique genetic traits and growth conditions of Hani terrace red rice may influence the structural properties of its starch, affecting its thermal behavior [33]. Additionally, the size and shape of the starch granules are also key factors, with larger or more regularly shaped granules typically exhibiting higher thermal stability [32]. Thus, the starch with a higher T_o_ required more energy to form a gel, more suitable for thermal processing under a high temperature. The starch isolated from organic brown rice (SOR08) exhibited the lowest T_o_, which could gelatinize easier than other starches. Variations in the onset temperature (T_o_), peak temperature (T_p_), and conclusion temperature (T_c_) of starch gelatinization can be attributed to differences in the distribution of amylopectin chains. Longer amylopectin chains tend to retard gelatinization due to their ability to form more stable, complex structures, which require higher temperatures to break down [34]. Conversely, shorter amylopectin chains, particularly those with a degree of polymerization ranging from 6 to 9, facilitate gelatinization. These shorter chains are less complex and more easily disrupted, allowing for quicker and more efficient gelatinization at lower temperatures [30]. Additionally, the branching pattern of amylopectin also plays a significant role. Amylopectin with more frequent branching tends to gelatinize at lower temperatures due to the reduced molecular order and increased water accessibility [35]. Therefore, the specific structural characteristics of amylopectin, including chain length and branching frequency, are critical determinants of the gelatinization properties of starch.

ΔH indicates the disruption of the double helix structure in amylopectin, giving an overall measure of crystallinity [36]. The ΔH values varied from 6.74 to 13.91 J/g, which is a similar range to previous studies on rice starch [8,23]. In starches with orthorhombic crystal structures, the double helical arrangements of amylopectin are tightly packed, forming crystalline regions. Hydrogen bonds are established both within these areas and between adjacent double helices, providing additional stability to the starch granules. The stability of this structure can be quantified by the ΔH value (enthalpy change), which represents the energy required to disrupt these hydrogen bonds. During the heating or gelatinization process of starch, these hydrogen bonds are broken, leading to the dissociation of the double helical structures and the disintegration of the crystalline regions. The variations in the ΔH values reflect the differences in the stability of the crystalline regions among different starch samples [32,37]. A higher ΔH value indicates stronger carbohydrate–water interactions and more stable and ordered crystalline zones formed during the hardening process upon heating, probably resulting from the degradation of endogenous amylase [38].

Thermogravimetric analysis was employed to assess the thermal stability of starch during heating. This stability is largely influenced by the intrinsic structural properties of starch, including its structure of the amylopectin and amylose in it, crystalline and amorphous regions, and the strength of hydrogen bonds within these areas [39]. The mass loss observed in the isolated starch samples from 20 °C to 200 °C during thermal analysis corresponds to the evaporation of water [40]. This weight reduction is indicative of the moisture content and the thermal stability of the starch’s hydration shell. Essentially, it reflects the interaction between starch granules and water molecules, and how this interaction withstands increasing temperatures [40]. The stability of this hydration shell is crucial, as it influences both the physical properties and the thermal behavior of the starch [40]. The significant weight loss at approximately 300 °C is a complex process of starch change involving the dehydration and depolymerization of the saccharide rings [41]. This stage of thermal decomposition is generally considered to be an important attribute in the study of starch degradation mechanisms, attributed to the degradation of amylose and amylopectin and reflecting the stability of ordered starch chains. The DTG curve in the thermal analysis indicates the rate of starch decomposition. The lower DTG value indicates a higher rate of starch decomposition [39]. Therefore, the lower temperature corresponding to the highest rate of starch decomposition indicates a weaker thermal stability of the starch. Thus, the starch isolated from Jasmine rice presented the weakest thermal stability, while the starch isolated from organic brown rice presented the strongest thermal stability. The final stage of thermal decomposition for starch occurs between approximately 350 °C and 600 °C, culminating in the carbonization of the sample [42]. This process results in the formation of a carbon-rich residue, often characterized by a blackened, charred appearance, reflecting the substantial breakdown and transformation of the starch’s organic components into a primarily carbon-based structure [42].

### 4.4. Pasting Profiles of Isolated Rice Starches

Pasting is a process that occurs during or subsequent to the gelatinization of starch [8]. This phenomenon involves the swelling and subsequent breakdown of starch granules when heated in water, leading to the formation of a paste [8]. As a crucial characteristic, the pasting profile of starch provides insight into the behavior of starch during thermal processing [8]. It serves as an indicator of the functionality and quality of rice and starch-based products, particularly in cooking applications [8]. These pasting characteristics are also instrumental in evaluating the potential of starch in applications such as texturizing agents. The comprehensive measurement process in a Rapid Visco Analyzer (RVA) encompasses several continuous phases of swelling: heating with a progressive increase in temperature, steadying at the peak temperature, subsequent cooling, and steadying at the final cooling temperature [8]. Key types of swelling characteristics include initial swelling, where granules start to absorb water; peak viscosity, indicating maximum swelling; breakdown, showing viscosity decrease due to granule disintegration; and setback, reflecting increased viscosity during cooling and tendency toward retrogradation. Additionally, pasting temperature marks the onset of noticeable swelling, and final viscosity indicates the stability of the gel after cooling [8]. These characteristics vary depending on the starch’s amylose and amylopectin contents, affecting its applications in various industries.

Pasting temperature (PT) is the temperature at the beginning of increasing viscosity and indicates the lowest temperature for heating the starch, ranging from 69.43 °C to 83.40 °C for isolated starches. The elevated pasting temperature (PT) observed in the starch isolated from Hani terrace red rice (SOR04) is indicative of its enhanced resistance to swelling, a characteristic that can be largely attributed to its higher crystallinity [43]. In starch granules, crystalline regions are pivotal in imparting thermal stability [44]. An increase in crystallinity leads to a corresponding increase in the granules’ resistance to thermal degradation [44]. This enhanced resistance manifests as a requirement for a higher temperature threshold to initiate the pasting process. Therefore, the higher crystallinity in the SOR04 starch granules is a key factor in its increased PT, reflecting its augmented resistance to swelling during thermal processing. Moreover, the PT was higher than the onset temperature (55.65 to 70.61 °C) of gelatinization determined by DSC, as the viscosity starts to increase only after the starch granules are thoroughly gelatinized [45]. 

With heating under increasing temperature, the viscosity increases, caused by the loss of free water, until it reaches the peak viscosity (PV) [46]. The PV reveals the degree of swelling and water-binding capacity of starch granules, correlated to the final texture of the starch product [24,47]. Starches isolated from glutinous rice varieties (SOR02 and SOR09) exhibited significantly lower peak viscosity (PV) values compared to those isolated from non-glutinous rice. This difference can be attributed to the higher amylopectin content in glutinous rice starch. Amylopectin, with its highly branched structure, does not form as rigid a gel network upon heating as amylose content [48,49]. Consequently, during heating, the starch granules in glutinous rice swell to form less rigid particles, particularly in the compact regions of the branched chains [48,49]. This results in a lower resistance to flow, leading to a lower peak viscosity. The starch isolated from organic brown rice (SOR08) exhibits the highest peak viscosity (PK). In the food industry, this suggests that such starches, taking longer to reach their peak viscosity, may require a more prolonged heat treatment to achieve the desired consistency and quality in the final product [46].

The trough viscosity (TV) of isolated starch denotes the minimum viscosity observed subsequent to the peak viscosity (PV) under a sustained high temperature [46]. This phenomenon results from the significant collapse of starch granules. Furthermore, the difference between TV and PV is referred to as breakdown viscosity (BD). During the BD stage, constant high temperature and continuous stirring cause the starch’s crystalline regions to melt, leading to water expulsion, the release of soluble starch polymers, and increased collisions among swollen granules [50]. BD is a parameter to assess the degree of stability during cooking and resistance to high-shear treatment when starch is used as a texturizing agent [5]. The starch isolated from Hani terrace red rice (SOR04) exhibited the lowest breakdown viscosity (BD) value, suggesting its higher resistance to shearing and heating conditions. This characteristic of SOR04 can be potentially attributed to its lower amylose content. The ratio of amylose to amylopectin in starch significantly influences its BD value, as amylose contributes to forming a more stable network during heating [51]. Starches with lower amylose contents tend to have fewer stable structures, impacting their breakdown viscosity [51]. Moreover, the starch isolated from Jasmine rice (SOR01) presented the highest BD value, which was correlated to its high PV value. Furthermore, the extent of breakdown can depend on starch type and quantity, the heating rate and temperature, and applied force [50]. The relationship between these factors and BD requires further exploration.

As the temperature decreases, viscosity increases progressively until reaching a final, stable value, a phenomenon termed retrogradation, attributed to the reassociation of amylose molecules. The disparity between the transitional viscosity (TV) and final viscosity (FV) is defined as the setback viscosity (SB), a critical determinant of the end-product’s texture [46]. The starch isolated from black rice (SOR03) exhibited the highest SB value, suggesting that SOR03 had a higher tendency to retrograde than others. The highest setback viscosity (SB) value observed in SOR03 can be attributed to its high amylose content. Higher levels of amylose are likely to contribute to an increased SB, as amylose tends to retrograde more readily during cooling, forming a denser and more rigid network. This enhanced retrogradation is a result of the amylose molecules’ tendency to reassociate and entangle upon cooling, leading to a firmer texture in the starch gel [52]. Therefore, the SB value of starch indicates how amylose molecules, leached out during gelatinization, interact and reassociate during cooling. This reassociation forms a more structured network, increasing viscosity and reflecting the extent of starch retrogradation [53].

Differences in pasting characteristics result from the amylose content, the distribution of amylopectin, and the degree of crystallinity [54]. Based on the determined pasting profiles, the isolated starch with high PV and BD values and low SB values indicates a good cooking quality of rice, corresponding to the starch isolated from Jasmine rice (SOR01) [50].

### 4.5. Digestibility of Rice Starch Based on In Vitro Model

Amylose has a firm molecular structure that packs its functional groups tightly, resulting in its lower tendency to digest [54]. Cooked starch with more amylose is less susceptible to digestive enzymes because of its distinct and ordered gelatinized structure, which slows down digestion within the gastrointestinal system [55]. Thus, the starches isolated from glutinous rice (SOR02 and SOR09), which contained lower amylose content, presented higher rapidly digestible starch (RDS) contents than those of non-glutinous starch; the current results are identical with a previous study [55].

During cooking, changes in the structural attributes of starch products occur mainly because of amylopectin. During the heating stage, the starch undergoes gelatinization, and the amylopectin becomes disordered, generating more sites for binding to digestive enzymes, which facilitates the digestion of starch [54]. Starches isolated from glutinous rice (SOR02 and SOR09) had a higher content of rapidly digestible starch as they contained more amylopectin, which facilitated digestion after gelatinization. However, during the cooling of the starch products, starch retrogradation occurs, and the amylopectin rearranges to generate a robust double helix structure, allowing the starch to resist binding to digestive enzymes [37]. Since the starch retrograded during the two-hour digestion, the starches isolated from glutinous rice (SOR02 and SOR09) had a high resistant starch content. Moreover, the starch isolated from black rice (SOR03) presented the highest content of slowly digestible starch, which is slowly hydrolyzed into glucose in the small intestine, considered as the desirable rice for digestion [56].

Moreover, the digestibility of starch is associated with the semi-crystalline structure [54]. The starch isolated from rice belongs to A-type starch, which is more susceptible to digestive enzymes than others, resulting from the existence of weak points in the structure [26]. 

### 4.6. PCA and Correlation Analysis of Starch Properties

Principal component analysis (PCA) was employed on the physicochemical properties of ten rice starch samples. The loading plot (Figure 5A) of PCA shows the relationship between starch properties parameters, and the score plot (Figure 5B) shows the similarities and differences among different varieties of starch. 

In the scores plot (Figure 5B), the clear delineation of the waxy and organic waxy rice starches indicates their similar starch properties. The starch from the glutinous rice (SOR02 and 09) possessed a higher swelling power and resistant starch content (Figure 5A,B). Starches isolated from Jasmine rice, Youzhan, and Wanniangong rice were within the same cluster since all of them belong to *Indica* species. The similarity of their characteristics may be related to their cultivation site located in the south of China. Moreover, starch from Hani terrace red rice possessed different characteristics, mainly in pasting temperature and enthalpy of gelatinization. Furthermore, Wuchang and organic Wuchang rice starch mainly exhibited different properties in thermal stability.

Based on the correlation analysis (Figure 5C), the amylose content of starch was associated with several parameters, as it largely determines the structure of the starch and its functional properties during pasting. The amylose content was positively correlated with the peak viscosity largely due to the linear nature of amylose molecules which can form a stronger and more stable gel network during the heating process, contributing to a higher viscosity. Moreover, during the cooling process, the amylose can interact with amylopectin and reform a network, which largely contributes to the viscosity and, thus, is positively related to the setback viscosity [57]. Furthermore, the amylose content was negatively correlated with the resistant starch content. Though a higher amylose content in starches is generally associated with an increased level of resistant starch, shorter branch chains of amylopectin can crystallize and resist digestion, increasing RS content [58]. For the swelling power, the branched structure of amylopectin allows for easier water penetration and swelling of the starch granule, leading to a greater swelling power. Conversely, the linear, more tightly packed nature of amylose tends to limit water penetration and, thus, the swelling capacity [59]. Thus, the amylose content was negatively correlated with the swelling power of the starches. The combination of plots elucidates the intricate relationships among the rice starch’s physicochemical properties, which are largely determined by the amylose content of starch.

The analyses presented in Figure 5A,B, alongside the correlation heatmap, collectively elucidate the intricacies of starch characteristics. The PCA underscores crystallinity’s profound influence on the starch matrix architecture, which, according to Wang and Ratnayake [60], is pivotal to a starch’s thermal and rheological behaviors. The heatmap, reinforcing these findings, suggests that the molecular composition of starch, particularly the amylose-amylopectin ratio, plays a crucial role in its pasting properties and digestibility [61]. Moreover, the correlation between peak and setback viscosities may be indicative of the starch’s retrogradation tendencies, a key factor in textural qualities of end products, aligning with the insights of Charles et al. [53]. The collective data point to the potential of starch source and processing diversity to affect functional properties, necessitating comprehensive characterization for tailored food application.

## 5. Conclusions

In the present study, the morphological, thermal, pasting, and digestive properties of starches from ten rice varieties were compared. All isolated rice starches showed an irregular polyhedral shape in the SEM and A-type starch characteristics in the XRD patterns of ten orthorhombic crystal structures. The starches isolated from different rice varieties showed different thermal behaviors during gelatinization. Both pasting and thermal properties showed that the starch isolated from Hani terrace red rice exhibited the highest stability under heating, which is suitable for cooking at high temperatures. In addition, the starch isolated from glutinous rice showed a higher swelling power and higher content of slowly digestible starch and resistant starch. The starch isolated from black rice showed a higher tendency to retrograde and may be the desirable variety of rice for blood sugar and body weight management due to its high slowly digestible starch content. In addition, PCA was performed to determine the importance of each attribute, while a correlation analysis was used to evaluate their mutual relationship. There is a correlation between amylose and several functional properties of starch, and, therefore, the amylose content may be an indicator of the application of starch in industry.

## Figures and Tables

**Table 1:** Different varieties of rice and their attributes.

Sample Code *	Common Name	Sub Species	Types of Rice	Place of Origin	Morphological Properties of Grains	Length–Width Ratio of Grain	Weight (g) of 1000 Grains
R01	Jasmine rice	Indica	Aromatic, non-glutinous	Kunming, Yunnan, China	White, thin, and long	3.97 ± 0.3 ^a^	19.27 ± 0.15 ^e^
R02	Waxy rice	Japonica	Glutinous	Mudanjiang, Heilongjiang, China	White, short, and round	1.56 ± 0.08 ^g^	18.45 ± 0.23 ^f^
R03	Black rice	Japonica	Non-glutinous	Mudanjiang, Heilongjiang, China	Black and long	2.14 ± 0.08 ^f^	18.55 ± 0.26 ^f^
R04	Hani terrace red rice	Indica	Non-glutinous	Honghe, Yunnan, China	Light red and long	2.37 ± 0.15 ^e^	25.14 ± 0.15 ^a^
R05	Youzhan rice	Indica	Non-glutinous	Dongguan, Guangdong, China	White, thin, and long	3.71 ± 0.27 ^b^	13.67 ± 0.45 ^h^
R06	Wuchang rice	Japonica	Non-glutinous	Harbin, Heilongjiang, China	White and short	2.14 ± 0.1 ^f^	19.98 ± 0.12 ^d^
R07	Wanniangong rice	Indica	Non-glutinous	Shangrao, Jiangxi, China	White, thin, and long	3.55 ± 0.22 ^c^	17.11 ± 0.18 ^g^
R08	Organic brown rice	Japonica	Non-glutinous	Suihua City, Heilongjiang, China	Beigh and short	2.12 ± 0.07 ^f^	21.85 ± 0.07 ^c^
R09	Organic waxy rice	Japonica	Glutinous	Chaoyang, Liaoning, China	White and short-round	1.56 ± 0.07 ^g^	19.20 ± 0.17 ^e^
R10	Organic wuchang rice	Japonica	Non-glutinous	Wuchang, Heilongjiang, China	White and long	2.87 ± 0.11 ^d^	22.59 ± 0.20 ^b^

The table displays the average value ± standard deviation and different letters. Statistically significant differences were found for the same column values (*p* < 0.05, *n* = 3). * The R in the sample code indicates the rice.

**Table 2:** XRD peaks analysis of various rice starch samples.

Samples	2*θ* (Degree)			
SOR01	15.099	17.184	17.875	22.974
SOR02	15.090	17.280	17.853	22.936
SOR03	15.265	17.321	18.131	23.275
SOR04	15.009	17.062	17.949	22.777
SOR05	15.194	17.091	18.012	23.005
SOR06	15.435	17.231	18.069	23.062
SOR07	15.377	17.290	18.116	23.181
SOR08	15.152	16.968	18.121	22.987
SOR09	15.065	17.219	17.849	22.891
SOR10	15.185	17.270	18.079	23.026

**Table 3:** The physicochemical attributes of isolated starch.

Sample Code	Moisture Content (%)	Color Characteristics			Amylose Content (%)	Crystallinity (%)	Swelling Power (g/100 g)	Water Solubility Index (g/100 g)
		L* (Lightness)	a* (Redness)	b* (Yellowness)				
SOR01	9.25 ± 0.06 ^a^	98.76 ± 0.01 ^b^	−1.01 ± 0.01 ^h^	1.06 ± 0.01 ^f^	19.84 ± 0.14 ^f^	34.13 ± 0.05 ^b^	36.47 ± 2.87 ^d^	9.8 ± 0.10 ^cd^
SOR02	8.91 ± 0.09 ^b^	98.56 ± 0.02 ^h^	−0.16 ± 0.01 ^c^	0.81 ± 0.01 ^i^	1.67 ± 0.01 ^j^	28.04 ± 0.64 ^d^	51.20 ± 0.24 ^a^	0.00 ^e^
SOR03	4.44 ± 0.03 ^f^	94.66 ± 0.01 ^i^	0.86 ± 0.01 ^a^	6.14 ± 0.01 ^a^	26.57 ± 0.31 ^a^	33.1 ± 0.26 ^c^	32.61 ± 0.85 ^e^	17.71 ± 1.38 ^a^
SOR04	3.88 ± 0.02 ^g^	96.27 ± 0.03 ^h^	0.67 ± 0.01 ^b^	3.46 ± 0.02 ^b^	13.95 ± 0.02 ^h^	32.89 ± 0.83 ^c^	40.96 ± 1.83 ^b^	9.36 ± 1.09 ^d^
SOR05	5.67 ± 0.06 ^d^	98.23 ± 0.06 ^f^	−0.37 ± 0.02 ^f^	1.43 ± 0.05 ^d^	23.54 ± 0.70 ^d^	25.33 ± 0.23 ^e^	38.87 ± 1.37 ^c^	10.93 ± 0.96 ^bc^
SOR06	5.96 ± 0.06 ^c^	98.45 ± 0.02 ^d^	−0.24 ± 0.01 ^d^	1.02 ± 0.02 ^g^	21.68 ± 0.10 ^e^	23.82 ± 0.44 ^f^	34.74 ± 0.25 ^ed^	16.76 ± 0.10 ^b^
SOR07	6.00 ± 0.05 ^c^	98.35 ± 0.10 ^e^	−0.16 ± 0.01 ^c^	1.07 ± 0.01 ^f^	19.09 ± 0.04 ^g^	28.32 ± 0.19 ^d^	41.13 ± 0.42 ^b^	10.02 ± 0.42 ^cd^
SOR08	4.72 ± 0.05 ^e^	98.13 ± 0.02 ^g^	−0.53 ± 0.01 ^g^	2.83 ± 0.02 ^c^	25.67 ± 0.13 ^b^	27.73 ± 0.09 ^d^	27.69 ± 0.12 ^f^	21.36 ± 0.76 ^ab^
SOR09	5.91 ± 0.17 ^c^	98.47 ± 0.01 ^d^	−0.22 ± 0.01 ^d^	0.96 ± 0.02 ^h^	2.55 ± 0.34 i	39.65 ± 0.33 ^a^	51.32 ± 0.3 ^a^	0.00 ^e^
SOR10	3.67 ± 0.10 ^h^	99.15 ± 0.02 ^a^	−0.29 ± 0.01 ^e^	1.38 ± 0.03 ^e^	25.08 ± 0.11 ^c^	34.08 ± 0.09 ^b^	34.35 ± 0.61 ^ed^	16.93 ± 1.05 ^b^

The table displays the average value ± standard deviation and different letters. Statistically significant differences were found for the same column values (*p* < 0.05, *n* = 3). SOR01, SOR02, SOR03, SOR04, SOR05, SOR06, SOR07, SOR08, SOR09, and SOR10 indicate starch isolated from Jasmine rice, waxy rice, black rice, Hani terrace red rice, Youzhan rice, Wuchang rice, Wanniangong rice, organic brown rice, organic waxy rice, and organic Wuchang rice, respectively.

**Table 4:** Pasting parameters of starch from different rice varieties.

Sample Code	PT (°C)	PV (cP)	TV (cP)	BD (cP)	FV (cP)	SB (cP)	Peak Time (Min)
SOR01	76.78 ± 0.03 ^c^	8402 ± 96 ^b^	1915 ± 11 ^g^	6487 ± 87 ^a^	3145 ± 6 ^g^	1230 ± 17 ^g^	4.73 ± 0 ^g^
SOR02	73.40 ± 0.44 ^e^	3603 ± 61 ^i^	1626 ± 27 ^i^	1977 ± 36 ^h^	2251 ± 45 ^h^	625 ± 22 ^h^	3.98 ± 0.04 ^i^
SOR03	74.70 ± 0.48 ^d^	7653 ± 187 ^c^	5215 ± 94 ^a^	2438 ± 160 ^g^	8138 ± 108 ^a^	2923 ± 15 ^a^	5.8 ± 0 ^a^
SOR04	83.40 ± 0.48 ^a^	4665 ± 23 ^g^	2877 ± 23 ^e^	1789 ± 36 ^i^	4764 ± 34 ^e^	1887 ± 13 ^d^	5.73 ± 0 ^b^
SOR05	78.83 ± 0.40 ^b^	7455 ± 71 ^d^	2044 ± 34 ^f^	5412 ± 77 ^c^	3386 ± 29 ^f^	1343 ± 34 ^f^	5.07 ± 0.07 ^f^
SOR06	74.15 ± 0.48 ^d^	7210 ± 65 ^e^	4385 ± 32 ^b^	2825 ± 91 ^e^	6815 ± 6 ^c^	2430 ± 32 ^c^	5.45 ± 0.04 ^d^
SOR07	78.25 ± 0.61 ^b^	8998 ± 34 ^a^	4070 ± 38 ^d^	4928 ± 8 ^d^	5697 ± 46 ^d^	1627 ± 19 ^e^	5.18 ± 0.04 ^e^
SOR08	72.53 ± 0.51 ^f^	6814 ± 207 ^f^	4202 ± 120 ^c^	2612 ± 88 ^f^	6958 ± 105 ^b^	2755 ± 15 ^b^	5.58 ± 0.04 ^c^
SOR09	69.43 ± 0.46 ^h^	4208 ± 49 ^h^	1560 ± 2 ^i^	2648 ± 51 ^f^	2204 ± 10 ^h^	644 ± 8 ^h^	3.4 ± 0 ^j^
SOR10	71.28 ± 0.08 ^g^	7675 ± 39 ^c^	1718 ± 11 ^h^	5957 ± 29 ^b^	3340 ± 16 ^f^	1622 ± 7 ^e^	4.13 ± 0 ^h^

The table displays the average value ± standard deviation and different letters. Statistically significant differences were found for the same column values (*p* < 0.05, *n* = 3). SOR01, SOR02, SOR03, SOR04, SOR05, SOR06, SOR07, SOR08, SOR09, and SOR10 indicate starch isolated from Jasmine rice, waxy rice, black rice, Hani terrace red rice, Youzhan rice, Wuchang rice, Wanniangong rice, organic brown rice, organic waxy rice, and organic Wuchang rice, respectively.

**Table 5:** Thermal properties of starch and digestive fractions of various starch samples.

Sample Code	T_o_ (°C)	T_p_ (°C)	T_c_ (°C)	ΔH (J/g)	RDS (%)	SDS (%)	RS (%)
SOR01	61.77 ± 0.46 ^c^	67.69 ± 0.33 ^b^	74.44 ± 0.33 ^c^	10.88 ± 0.62 ^b^	47.63 ± 0.68 ^d^	52.23 ± 0.68 ^d^	0.13 ± 0.03 ^e^
SOR02	58.83 ± 0.24 ^d^	67.58 ± 0.12 ^b^	78.81 ± 0.27 ^b^	11.13 ± 0.82 ^b^	74.32 ± 0.45 ^a^	23.41 ± 0.37 ^g^	2.27 ± 0.08 ^a^
SOR03	55.85 ± 0.55 ^g^	62.47 ± 0.44 ^de^	69.06 ± 0.14 ^ef^	10.89 ± 0.70 ^b^	39.80 ± 0.32 ^g^	59.48 ± 0.24 ^a^	0.72 ± 0.11 ^d^
SOR04	70.61 ± 0.48 ^a^	75.61 ± 0.15 ^a^	81.04 ± 0.41 ^a^	13.91 ± 0.69 ^a^	54.39 ± 0.65 ^c^	44.97 ± 0.66 ^e^	0.64 ± 0.01 ^d^
SOR05	61.37 ± 0.47 ^c^	67.90 ± 0.35 ^b^	74.11 ± 0.24 ^c^	9.14 ± 0.48 ^c^	43.63 ± 0.83 ^e^	54.92 ± 0.83 ^c^	1.46 ± 0.16 ^b^
SOR06	56.03 ± 0.22 ^g^	61.86 ± 0.64 ^ef^	69.48 ± 0.42 ^e^	9.37 ± 0.24 ^c^	44.13 ± 1.01 ^e^	55.15 ± 0.97 ^c^	0.72 ± 0.05 ^d^
SOR07	63.03 ± 0.33 ^b^	67.95 ± 0.16 ^b^	73.36 ± 0.07 ^d^	7.63 ± 0.19 ^d^	47.34 ± 1.10 ^d^	51.54 ± 1.14 ^d^	1.12 ± 0.08 ^c^
SOR08	55.65 ± 0.03 ^g^	61.52 ± 0.49 ^f^	67.89 ± 0.45 ^g^	6.96 ± 0.13 ^ed^	41.28 ± 1.23 ^fg^	57.58 ± 1.13 ^b^	1.14 ± 0.11 ^c^
SOR09	56.89 ± 0.19 ^f^	64.24 ± 0.24 ^c^	74.23 ± 0.80 ^c^	8.63 ± 0.06 ^c^	72.43 ± 1.65 ^b^	25.27 ± 1.67 ^f^	2.3 ± 0.05 ^a^
SOR10	57.95 ± 0.25 ^e^	63.06 ± 0.41 ^d^	68.71 ± 0.07 ^f^	6.74 ± 0.10 ^e^	41.63 ± 1.10 ^f^	57.03 ± 1.08 ^b^	1.33 ± 0.04 ^b^

The table displays the average value ± standard deviation and different letters. Statistically significant differences were found for the same column values (*p* < 0.05, *n* = 3). SOR01, SOR02, SOR03, SOR04, SOR05, SOR06, SOR07, SOR08, SOR09, and SOR10 indicate starch isolated from Jasmine rice, waxy rice, black rice, Hani terrace red rice, Youzhan rice, Wuchang rice, Wanniangong rice, organic brown rice, organic waxy rice, and organic Wuchang rice, respectively.

## Data Availability

Data is contained within the article.
